# General Practice Community Pharmacist Consultation Service: an exploratory patient survey

**DOI:** 10.3399/BJGPO.2024.0204

**Published:** 2025-04-24

**Authors:** Julia Gauly, Catherine Grimley, Jeremy Dale, Paramjit Gill, Helen Atherton

**Affiliations:** 1 Warwick Medical School, University of Warwick, Coventry, UK; 2 Primary Care Research Centre, University of Southampton, Southampton, UK

**Keywords:** Community pharmacy, consultation, general practice, GP CPCS, survey

## Abstract

**Background:**

The General Practice Community Pharmacist Consultation Service (GPCPCS) was established to allow patients with certain minor illnesses to be referred to a community pharmacy for assessment and treatment.

**Aim:**

To explore patients’ experiences of the GPCPCS.

**Design & setting:**

An online survey in two regions of England.

**Method:**

Twenty-five general practices invited patients to take part in an exploratory survey. Descriptive statistics were used for the analysis.

**Results:**

The response rate was 5.1% (72/1423). Prior to contacting their general practice, 14.1% (9/64) of patients had tried to speak to a pharmacist. Most responders accepted the GPCPCS referral (77.3%, 51/66); received a pharmacy consultation on the same day (80.0%, 40/50); and were largely satisfied with the amount of time the pharmacist spent with them (82.5%, 33/40), the consultation format (68.3%, 28/41), and the privacy provided during the consultation (80.9%, 38/47). However, most responders (56.5%, 39/69) felt poorly informed by the general practice on why they were being advised to speak to a pharmacist and did not feel that it was appropriate that they had been advised to speak to a community pharmacist (54.2%, 39/72). Only 33.3% (16/48) felt that their consultation fully met their health needs and 27.1% (13/48) of patients described being re-referred from pharmacy back to their general practice.

**Conclusion:**

In this exploratory study patients were largely accepting of the GPCPCS. Improvements in terms of explaining GPCPCS to patients, selecting patients appropriate for referral to the service, and the appointment process may be of benefit.

## How this fits in

This study is the first independent research on patients’ experiences of the General Practice Community Pharmacist Consultation Service (GPCPCS), which was introduced to refer patients with certain minor illnesses from general practices to pharmacies in order to free up GPs’ time for patients with more serious or complex conditions. Our findings suggest that the service, which has been incorporated into Pharmacy First since January 2024, works well for most patients, but that service specifications are not met in all cases and that improvements in terms of explaining GPCPCS to patients, identifying appropriate patients, and the appointment process may need to be considered. While our findings cannot be generalised due to the small sample size, they are useful to inform service development and future research.

## Introduction

Some patients in England attend general practice with clinically divertible, low-acuity conditions that could be safely transferred to a community pharmacy.^1^ It is estimated that they account for 6% of all GP consultations, that is, 20.4 million appointments per year.^2^ Various pharmacy schemes have been introduced to take pressure from general practices.^3^ These include the General Practice Community Pharmacist Consultation Service (GPCPCS), which was introduced in 2020^4^ and incorporated into the most recent scheme ‘Pharmacy First’ in January 2024.^5^ It was the first service allowing general practices to formally refer patients to the community pharmacy.

Under the GPCPCS scheme, reception staff offer patients contacting their general practice for certain minor illnesses a referral for a community pharmacy consultation.^6^ Table 2 shows the range of health concerns that patients could be referred for as part of the GPCPCS. If the patient agrees, an online referral is made to the patient’s preferred pharmacy for a consultation delivered over the phone or in a private consultation room at the pharmacy. While patients can still be referred from general practices to community pharmacies under ‘Pharmacy First’, the scheme also allows patients to self-refer for conditions including sinusitis, sore throat, earache, impetigo, shingles, and uncomplicated urinary tract infection in women.^7^

In the context of 11 600 pharmacies in England, the initial number of referrals for the GPCPCS was low with only 7000 referrals per week in 2022.^8,9^ As diverting patients to pharmacies remains a key element of health policy,^5^ and exploring patient experience is important to gain insights from patients on how to improve care,^10^ one aspect of exploring this research area is to understand patients’ views of being referred by their general practice to the pharmacy. This study aimed to explore this.

## Method

This exploratory survey of patients who had been referred to the GPCPCS was conducted between December 2022 and October 2023.

### Study setting

The study was set in two English regions.

### Sample and recruitment

Eligible patients were individuals aged ≥16 years who were documented in the patient record system as having been offered a GPCPCS referral for themselves or someone they care for within the past 3 months prior to being invited to participate in the survey.

Clinical Research Networks (CRN) in the North West and the West Midlands of England supported the recruitment of practices that were signed up for GPCPCS. CRN facilitators also supported participating practices in sending out invitations to participate via text messages to eligible patients iteratively over the recruitment period. A reminder text message was sent after 2 weeks.

### Data collection

The survey was anonymous and completed online. Informed consent was obtained from patients before the beginning of the survey. Data was collected using Qualtrics.^11^

#### Survey instrument

The text message to patients included a practice code, which responders were asked to indicate at the beginning of the survey. Questions specific to GPCPCS were informed by reports on GPCPCS,^6,11^ leads of the GPCPCS in the West Midlands, research team members, and feedback from the public. Questions on patients’ experiences of visiting the pharmacy and the pharmacy consultation included those from previously published surveys.^10,12,13^ Validated demographic questions were from previously published surveys.^10,14–16^ The survey originally comprised 37 questions (see Supplementary Survey S1). To increase the response rate, survey length was shortened to 20 questions 2 months into the recruitment period^17^ by omitting questions on general pharmacy usage and details about individual consultations (see Supplementary Survey S2).

#### Survey piloting

Four members of the public with experience in pharmacy and general practice reviewed the survey for clarity and comprehensiveness, and their feedback was incorporated. Cognitive interviews were then conducted with an additional four participants,^10^ leading to further refinement of the survey instrument based on their insights. The survey was reviewed in a public and patient involvement meeting and feedback incorporated. Additionally, cognitive interviewing (*n* = 3)^10^ was conducted before finalising the survey.^16^

### Data processing and analysis

Data were exported from Qualtrics^12^ into Excel and cleaned. Data from incomplete surveys were included if a valid practice code was entered and more than three answers were completed. Descriptive statistics were used to analyse patients’ experiences, usage, and outcome of GPCPCS and willingness to use GPCPCS again. Free text data were categorised using an abductive approach,^13^ supported by quotes with demographic characteristics. Responses to questions included in both the longer and shorter survey were analysed together, while those questions only included in the long survey were analysed separately. Stata (version 15) was used for the analysis.

## Results

In total, 142 general practice were invited and 25 general practices participated. Of those, 23 general practices were from the West Midlands and two general practices from the North West. They sent invitations to 1423 patients recorded as having been referred to the GPCPCS. The response rate was 5.1% (72/1423).

Out of the 72 responses (64 responses on the full-length survey version and eight on the shortened survey version) were included. Seven of the eight responses on the shortened survey and 53 of the 64 responses on the full-length survey were fully complete. Table 1 shows an overview of responders’ demographic characteristics.

**Table 1.** Responders’ demographic characteristics

	*n*	**%**
**Total**	72	100
**Gender**		
Female	45	62.5
Male	14	19.4
Non-binary	1	1.4
Prefer not to say	2	2.8
Unknown	10	13.9
**Age group, years**		
18–24	4	5.6
25–34	13	18.1
35–44	9	12.5
45–54	11	15.3
55–64	8	11.1
65–74	13	18.1
75–84	3	4.2
Prefer not to say	1	1.4
Unknown	10	13.9
**Ethnicity**		
White: English, Welsh, Scottish, Northern Irish	51	70.8
White: Any other White background	3	4.2
Asian/Asian British	3	4.2
Black/African/Caribbean/Black British	1	1.4
Other ethnic group: Arab	1	1.4
Prefer not to say	3	4.2
Unknown	10	13.9
**Employment status**		
In full-time paid work (≥30 hours)	24	33.3
Part-time paid work (<30 hours)	7	9.7
Unemployed	2	2.8
Permanently sick or disabled	5	6.9
Fully retired from work	10	13.9
Looking after the family or home	3	4.2
Prefer not to say	9	12.5
Unknown	12	16.7

Most responders contacted their general practice about their own health concerns (93.8%, 60/64) rather than for someone that they care for (6.3%, 4/64). As shown in Table 2, responders described that they had been referred to the pharmacy for a variety of health concerns.

**Table 2.** Range of health concerns that responders reported for being referred for to GPCPCS

Patients self-reported health condition(s)/health concern(s)^a^	*n*	%
24-hour blood pressure monitoring^b^	*1*	1.6
Acne, spots and pimples	1	1.6
Allergic reactions	4	6.3
Ankle and foot pain or swelling	1	1.6
Bites or stings (insect or spider)	2	3.1
Cold or flu	4	6.3
Cough	5	7.8
Diarrhoea	3	4.7
Ear discharge or ear wax	2	3.1
Earache	1	1.6
Emergency contraception^b^	*2*	3.1
Eye: red, irritable, sticky, watery or eyelid problem	2	3.1
Headache	5	7.8
Jaw pain^b^	*1*	1.6
Knee or lower leg pain	2	3.1
Lower back pain	2	3.1
Mastitis^b^	*1*	1.6
Mouth rash^b^	*1*	1.6
Nasal congestion	4	6.3
Not specified^b^	*4*	6.3
Other eye or ear problem^b^	*2*	3.1
Skin, rash	6	9.4
Sleep difficulties	2	3.1
Sore throat	5	7.8
Stomach issues (vomiting and nausea)^b^	*1*	1.6
Temperature^b^	*1*	1.6
Tiredness	2	3.1
UTI/cystitis^b^	*7*	10.9
Vaginal itch or soreness	2	3.1
Don’t know/not sure	10	15.6

^a^Health concern(s)/condition(s) for which general practices can refer to but were not indicated by responders were: Athlete’s foot, blisters, constipation, hair loss, hearing problems or blocked ear, hip, thigh, or buttock pain or swelling itch, lower limp pain or swelling , mouth ulcer, rectal pain, scabies, shoulder pain, toe pain or swelling, vaginal discharge, vomiting, wound problems (management with dressing) and wrist, hand or finger pain or swelling. ^b^Multiple responses could be indicated here; *n* = 64. Health concern(s)/condition(s) which were not on the official GPCPCS list but indicated by patients under ‘Other, please specify’. UTI = urinary tract infection.

Prior to contacting their general practice, responders had taken a range of actions to address their health concern(s) (see Table 3) but only 14.1% (9/64) had tried to speak to a pharmacist.

**Table 3.** Actions by responders take prior to contacting the general practice

Total^a^	*n*	**%**
‘Asked for advice from a friend or family member‘	17	26.6
‘Tried to treat myself/the person I was making this appointment for (for example with medication)’	32	50
‘Spoke to a pharmacist’	9	14.1
‘Called an NHS helpline, such as NHS 111’	4	6.3
’Tried to get information or advice elsewhere (from a non-NHS service)’	6	9.4
’Used an online NHS service (including NHS 111 online)’	11	17.2
’Used a non-NHS online service, or looked online for information’	8	12.5
’Contacted or used another NHS service’	3	4.7
’I did not try to get information or advice’	10	15.6

^a^Multiple responses could be provided for this question, *n* = 64.

The majority of responders agreed that their health concern could be considered as minor (55.2%, 32/58). Participants’ thoughts on whether it was appropriate that they had been advised to speak to a pharmacist were mixed: 45.8% (33/72) felt it was appropriate, 29.2% (21/72) were unsure, and 25.0% (18/72) felt that it was not appropriate. The majority of responders felt poorly informed by the general practice on why they were being advised to speak to a pharmacist (56.5%; 39/69), the range of health concerns for which their general practice could refer them (67.2%, 43/64), what would happen next should they decide to speak to a pharmacist (55.7%, 34/61), and what to expect from a pharmacy consultation (63.8%, 44/69). When being referred, most responders agreed to speak to a pharmacist (77.3%, 51/66). Of the responders, 80.0% (40/50) received a pharmacy appointment on the same day as their referral. Among them, 50.0% (20/40) had a phone consultation, while the other 50.0% (20/40) attended a face-to-face consultation.

Nevertheless, several responders indicated in the free-text comments that they experienced difficulties in obtaining a consultation because there was no pharmacist available, or responders were not being contacted by the pharmacy (Table 4).

**Table 4.** Categorised free text comments with example quotes

Category	Examples from sample
**Reasons to refuse referral**	**Lack of ability to perform check up/prescribe antibiotics**
	*‘Pharmacists can’t perform a check-up or prescribe antibiotics‘* (patient identifier number [PIN] 11 Female, Arab,18–24 years)
	**Inconvenience**
	*‘If I’m already at the GP, where they can inform of the same thing that the pharmacy can, why would I travel to the pharmacy for the same advice?‘* (PIN 57, Female, Asian, 18–24 years)
**Experiences of being re-referred to GP**	**No appointments left at general practice**
	*‘I was referred back to the GP but there were no appointments left that day by the time I spoke to a pharmacist so I went to a walk in centre and waited 3 hours to be seen. A lot of wasted NHS time and services. Three places involved when it only needed one.’* (PIN 64, Female, White, 25–34 years)
**Negative experience with pharmacist consultation**	**Inability of the pharmacist to deal with health concern**
	*‘He was so confused he didn’t seem to know what he was doing. I was given a nasal spray which was basically useless. It rather made it worse on the first use.’* (PIN 71, Female, Black, 25–34 years old)
	**Un-caring pharmacist**
	*’Didn’t look at my daughter, didn’t examine her just advised to buy over counter medication, a waste of time really.’* (PIN 20, Female, White, 35–44 years)
	**Pharmacist too busy**
	*’Pharmacist was really too busy.’* (PIN 74, Female, White, 65–74 years)
	**Conflicting advice**
	*’The problem is not with my general practice, but with conflicting advice from the pharmacies.’* (PIN 68, Female, White, 65–74 years)
**Service improvement ideas**	**Ensure that pharmacist is available**
	*‘Make sure that the pharmacist is even there!! Mine wasn’t. Had to find and ring around for another one. Took 8 hours to find one. Very disappointed.’* (PIN 37, Female, White, 45–54 years*)*
	**Ensure that pharmacist is trained to deal with the health concern that patient is being referred to**
	*’GP appears not to have been aware that pharmacy unable to help me on my specific circumstances.’* (PIN 39, Male, White, 35*–*44 years)
	**Provide more explanation from their general practice on the GPCPCS process**
	*’Overall, I think it’s a good idea, however I think it needs a bit more work as to explanation from the GP as to what will happen.’* (PIN 40, Female, Prefer not to say, 45–54 years*)*
	**Provide the option to have a telephone consultation**
	*’I also feel it’s unnecessary to have a face to face appointment, a telephone appointment is more than adequate which is what the GP said would happen but the pharmacy insisted I came to the pharmacy which was quite inconvenient.’* (PIN 40, Female, Prefer not to say, 45-54 years old)
	**Screen patients more carefully before referring**
	*‘A questionnaire about health problems to be filled in by patient, before being referred to the pharmacist.’* (PIN 61, Male, White, 65–74 years)
	**Give pharmacists authority to prescribe stronger medication**
	*‘There needs to be a better GP system of them seeing patients I would even pay as I have wasted ton of money and time and irritating condition to end up seeing a GP who within 5 minutes diagnosed what it was and the foam spray cleared everything up in a matter of days. Or give the pharmacist more authority to prescribe stronger meds.’* (PIN 5, Male, White, 35–44 years)
	**Use medically trained staff for triage**
	*’The receptionists are not medically trained so should not fob you off with speaking to a pharmacist.’* (PIN 24, Female, White, 45–54 years)

Responders (72.1%, 31/43) indicated that they had been contacted by the pharmacy (39.5%, 17/43) or contacted the community pharmacy themselves (32.6%, 14/43) within 3 hours after being referred.

Nearly a one-quarter of responders (22.7%, 15/66) reported declining a pharmacy consultation. The reasons given included feeling that it was inappropriate for a pharmacist to address their health concern(s) (35.3%, 6/17) or deciding to wait and see if their symptoms would resolve (11.7%, 2/17).

In the free-text comments, responders also expressed beliefs about pharmacists lacking the scope of practice to meet their requirements and about the inconvenience of attending the pharmacy (see Table 4). Most of those who refused the referral felt that nothing could have changed their decision (86.7%, 13/15). The majority of them were provided with a GP appointment instead (60.0%; 9/15).

Of those who saw a pharmacist, most were satisfied with the amount of time the pharmacist spent with them (82.5%, 33/40), which they generally described as lasting <10 minutes (62.5%, 25/40). Most (68.3%, 28/41) were also satisfied with the consultation format (face-to-face or phone). Of those who had a face-to-face consultation, most described having it in a private consultation room (81.8%, 18/22) and felt satisfied with the level of privacy provided (81%, 38/47). Consultation outcomes varied, with 27.1% (13/48) being referred back to their GP (see Table 5). One of the responders who was referred back to the general practice found that there were no appointments left by that time (see Table 4).

**Table 5.** Consultation outcome

Consultation outcome	*n*	**%**
**Total**	48	100
Advice given (verbal, printed and self-care)	10	20.8
Purchase of a prescription-free product (over-the-counter product)	13	27.1
Referral to another pharmacy	1	2.1
Referral to your GP	13	27.1
Referral to A&E	1	2.1
Advised to call 111^a^	1	2.1
Antibiotics provided^a^	1	2.1
Treatment given free of charge^a^	2	4.2
Prescription purchased^a^	1	2.1
Other (outcome not indicated by responders)	5	10.4

a Consultation outcomes which were not on the official GPCPCS list but indicated by patients under ‘Other, please specify’.

Only 33.3% (16/48) felt that their consultation fully met their health needs. Nevertheless, 54.2% (26/48) thought that the pharmacist’s ability to answer their questions was excellent or very good. In the free-text comments some responders reported negative experiences with the consultation; for example, feeling that the pharmacist did not seem competent to deal with their health concerns (see Table 4). While 38.3% (18/47) would be likely to attend the pharmacy first next time, 44.7% (21/47) were unlikely to do so, and 17.0% (8/47) were undecided.

A total of 15 responders (20.8%, 15/72) shared comments on how the GPCPCS could be improved. For instance, they highlighted the importance of having a pharmacist available who is adequately trained to address the health concerns for which they were referred to the pharmacy. They also wanted more explanation from their general practice on the GPCPCS process (see Table 4).

## Discussion

### Summary

This study is the first academic evaluation of patients’ experiences with the GPCPCS scheme. Most responders accepted their general practice’s referral to a pharmacy, received a same-day pharmacy consultation, and were largely satisfied with the consultation’s mode and timing and the pharmacists’ ability to answer questions. Most who refused the referral indicated that nothing could have changed their mind, and most had been provided with a GP appointment instead. Conversely, 27.1% of those who accepted a GPCPCS referral were subsequently referred back to their general practice. Widespread elements of the service were felt to need improvement. Most responders did not feel well informed about GPCPCS, the processes involved, or what to expect from the referral and pharmacy consultation. Some experienced difficulties in getting their consultation. There were mixed views on whether it was appropriate for their problem to be referred to a pharmacist and whether the pharmacy consultation met their health needs. There were also mixed views on whether they would use GPCPCS in the future.

### Strengths and limitations

This is an under-researched topic, despite its importance in current NHS policy in the UK. Since GPCPCS has been incorporated into Pharmacy First, the findings will be important for those delivering this new advanced pharmacy service. The response rate was 5.1%, which is low for patient surveys that recruit via general practice. This means that the findings from our survey cannot be generalised. The low response rate may indicate that many patients who practices thought were eligible to participate in the survey either did not recall being signposted to GPCPCS, recalled the signposting but did not pursue it, or were not interested in completing a survey on this topic. Previous research has shown that community pharmacies are a key resource for tackling health inequalities.^14^ For this exploratory study we did not include sociodemographic questions that would have allowed us to identify and explore the experiences with the GPCPCS of marginalised and vulnerable groups such as for example people experiencing homelessness, drug and alcohol dependence, vulnerable migrants, Gypsy, Roma and traveller communities, sex workers, people in contact with the justice system who are more likely to experience inequalities.^15^ This needs to be recognised as another limitation of the study.

### Comparisons with existing literature

In line with data from Solihull, more than one-quarter of those who reported having had a GPCPCS consultation stated that they had been referred back to their general practice.^16^ This indicates that the patient need was not adequately assessed, and it is wasteful of patient time and NHS resources. That pharmacy initiatives can mean that patients might attend both community pharmacy and general practice, and thereby increase GP workload was recognised in a recent short report on community pharmacy and general practice collaboration and integration.^17^

Our finding might also suggest that practice reception staff need more training on triaging patients and/or clearer guidance. Previous research has shown that non-clinician telephone triage can be problematic.^18,19^ A recent survey from England found that only 25.7% had received training on basic triage.^20^ A workshop on the GPCPCS concluded that general practice reception staff should be provided with more support on the management of referral cases.^4^ As of January 2024, more than 10 000 out of 11 600 pharmacies in England were signed up for the referral scheme.^9,21^ However, a survey by the National Pharmacy Association (NPA) reported that three-quarters of pharmacies are not getting regular referrals from general practices,^21^ which indicates that the triage system does not seem to work to the extent that the NHS had anticipated.

Our study found that many patients felt that they lacked information about the GPCPCS process. Receptionists have a key role in facilitating patient awareness regarding new approaches in primary care.^22^ However, since general practices are independent organisations, budgetary and time constraints can mean that receptionist training is sometimes overlooked, suggesting a need for this to be addressed as part of the implementation of the GPCPCS and similar schemes.^23^

Only 14.1% of responders had tried to speak to a pharmacist prior to contacting their general practice. This might indicate that people lack awareness of pharmacy services, something that has been found previously in the UK.^24,25^ Some responders indicated that there was no pharmacist available at the time that they attended. The lack of a pharmacist and insufficient staffing levels have been shown to be a barrier to pharmacy services previously.^9,26^

A recent survey conducted by Ipsos on behalf of NHS England found that most pharmacy users were able to get what they needed.^27^ However,^28^ responders in our survey had mixed views on whether their needs were met through the pharmacy consultation. It is not clear whether responders in the survey by Ipsos included users of the GPCPCS, which might explain the difference in experience on whether health needs were met. However, with 23 new Patient Group Directions (PGDs) introduced under Pharmacy First,^29^ which allow pharmacists to give antibiotics for seven conditions following a consultation, it is possible that pharmacists will be able to address patients’ health needs more sufficiently.^30^

### Implications for research and practice

More training, guidance, and resources for receptionist staff might be required to equip them to identify patients for referral to GPCPCS and explain the process to patients. Awareness of the pharmacists’ role and the referral pathway needs to be increased among both professionals and the public. General practices could share information about the referral pathway on their websites and through their interactive voice response systems. General practices and pharmacies could liaise more to understand the capacity of local pharmacies to ensure that those patients who are referred to a pharmacy have a pharmacist available. Our findings suggest that not all consultations take place in a private consultation room. Service specifications of the GPCPCS and Pharmacy First require face-to-face consultations to be delivered in a consultation room.^6,31^ Compliance checks are needed to ensure consistency in service provision.

In conclusion, more research is needed on patients’ experiences of minor illness referral schemes, including Pharmacy First. Future research should consider recruiting patients prospectively, such as immediately following a pharmacy consultation, and including qualitative approaches to gain a more comprehensive understanding of the subject. Future research should also ensure that marginalised and vulnerable groups are included in research on pharmacy services to understand how they are contributing to addressing health inequalities. Given that the referral rates have been low it is also important to explore the barriers to referring patients for minor illnesses from the perspectives of GP and reception staff.

## Data

The dataset relied on in this article is available from the corresponding author on reasonable request.
